# Impact of AI-generated characters on visual attention and prefrontal cognitive responses across age and image type conditions: using fNIRS and eye-tracking

**DOI:** 10.3389/fnhum.2026.1723608

**Published:** 2026-04-13

**Authors:** Jaechang Cha, Nakyung Lee, Seongdae Kim

**Affiliations:** 1 Department of Nano Technology and Advanced Materials Engineering, Sejong University, Seoul, Republic of Korea; 2 School of Design Convergence, Hongik University, Sejong, Republic of Korea; 3 Department of Biohealth Convergence Open Sharing System, Hongik University, Seoul, Republic of Korea

**Keywords:** age conditions, AI-generated characters, eye-tracker, fNIRS, image type conditions

## Abstract

With the rapid expansion of the digital environment, content employing Artificial Intelligence (AI)-Generated Characters has surged, underscoring the importance of establishing emotional rapport with users. This study employed an eye-tracker and functional near-infrared spectroscopy (fNIRS) to examine differences in users' visual attention and prefrontal cognitive responses across age conditions and image type conditions, using objective physiological and behavioral metrics. This study recruited 24 healthy university students and presented 18 static facial images that combined six age conditions ranging from infant to elderly with three image type conditions comprising real, two-dimensional (2D), and three-dimensional (3D) representations. Visual attention was measured with an eye-tracker using total duration of fixations, average duration of fixations, number of fixations, and average pupil diameter, while prefrontal cognitive responses were recorded with fNIRS as changes in oxygenated hemoglobin (HbO) concentration. When participants viewed images in the 3D condition, the middle-aged condition showed a significantly greater average pupil diameter than the other age conditions (*p* = 0.0031). Similarly, when participants viewed middle-aged condition images, the 3D condition yielded a significantly greater average pupil diameter than the other image type conditions (*p* = 0.0215). In the fNIRS data, child stimuli exhibited higher HbO concentration than the other age conditions at Channel 5 in the right Dorsolateral Prefrontal Cortex (DLPFC; *p* = 0.003) and at Channel 19 in the left DLPFC (*p* = 0.038). Regarding image type conditions, Channel 35 in the left DLPFC showed higher HbO for 2D and 3D than for real (*p* = 0.006). Age conditions and image type conditions significantly affected visual attention and prefrontal cognitive responses. This suggests that visual stimuli influence not only simple preference but also users' emotional response and broader cognitive processing. Furthermore, this study's integrated approach, combining an eye-tracker and fNIRS, can provide practical evidence for user experience–based AI content and interface design.

## 1 Introduction

Artificial Intelligence (AI)-Generated Characters are human-like virtual persons produced by generative artificial intelligence, and under appropriate conditions they can complement or replace human roles in advertising, brand marketing, social media, and customer service, increasing consumers' brand trust and advertising effectiveness (Liu and Lei, 2025). Owing to their scalability, consistency, and capacity for personalization, AI-Generated Characters are emerging as a core tool in digital communication and user experience design. The commercial success and social acceptance of such characters depend less on technical perfection than on consumers' perceptions and affective responses, specifically whether qualities of liking, trust, and intimacy within the user experience translate into sustained engagement and brand loyalty.

Such emotional rapport can arise through multiple pathways, but visual information plays a pivotal role in human cognition. Vision dominates the acquisition of environmental information and early judgments, and gaze and visual information can actively pull decision outcomes, including choice and value comparison (Shimojo et al., 2003). Rather than treating the appearance design of AI-Generated Characters as a mere aesthetic element, it is crucial to consider how it can elicit emotional rapport with users and, more specifically, which design elements actually induce greater liking and engagement. These issues are difficult to adjudicate with self-report alone.

In response, recent studies have actively introduced physiological and neuro-based measurement techniques, such as functional Near-Infrared Spectroscopy (fNIRS), electroencephalography (EEG), and an eye-tracker, into the design of AI-Generated Characters and the analysis of user responses to address these limitations (Zhang et al., 2023). In practice, the most widely used eye-tracker employs a video-based pupil–corneal reflection method, estimating ocular rotation and on-screen gaze position from the relative positions of the pupil center and the corneal reflection captured by the camera (Duchowski, 2017). fNIRS is a noninvasive technique that measures cerebral hemodynamic responses and interprets increases in oxygenated hemoglobin (HbO) as an index of activation in specific brain regions. Measurements are obtained by placing light sources and detectors at fixed separations; each source–detector pair constitutes a channel. Because each channel maps to a specific location on the cortical surface, array layouts are planned at the design stage to align with the researcher's regions of interest (Scholkmann et al., 2014). These devices capture users' visual attention and brain activity in real time, converting internal emotional and cognitive responses into objective metrics and thereby offering high practical utility. In particular, an eye-tracker yields gaze position and pupil responses, whereas fNIRS indexes changes in HbO within the prefrontal cortex. This measurement framework sensitively detects differences in responses as a function of stimulus characteristics.

Age is a primary determinant of users' emotional response and liking. Consistent with Baby Schema theory, infantile facial features elicit automatic caregiving responses and elevate liking, indicating that perceived age systematically shapes emotional response and social evaluation beyond aesthetics (Lorenz, 1943). In advertising and media communication contexts, similar principles have been observed: in public-interest and charity advertising, images of infants or children have repeatedly been reported to elicit greater empathy and higher donation intentions than images of adults (Burt and Strongman, 2005). Image type is likewise a key variable shaping users' perception and acceptance. Recent work reports that faces newly generated by generative AI are perceived as more trustworthy than real faces, and that people often fail to accurately distinguish the two types (Nightingale and Farid, 2022).

In general, liking and engagement manifest as fluctuations in emotional arousal immediately after a stimulus. A representative metric indexing this response is pupil diameter. Psychology and physiology have consistently reported that higher levels of emotional arousal are accompanied by greater pupil dilation (Bradley et al., 2008). Such physiological metrics provide objective cues for assessing the degree of liking and engagement toward specific visual stimuli. In evaluating and selecting visual stimuli, cognition and self-regulation operate jointly, and changes in prefrontal cortex activity play a key role. In particular, the Dorsolateral Prefrontal Cortex (DLPFC) is associated with attentional control and decision-making among higher cognitive functions and is engaged when users move beyond merely seeing to cognitively evaluating and judging stimuli (Kroger et al., 2002; Owen, 1997).

Prior studies have increasingly employed physiological and neuro-based measurement devices to test the effects of content design and visual stimuli. Within this stream of work, the central question is what actually drives users' liking and engagement. In studies comparing video stimuli that elicit emotional rapport with those that do not, peak pupil dilation was larger under the empathy condition, which has been reported as a metric indicating that viewers were more immersed in the narrative and emotionally engaged (Zhang et al., 2022). Thus, pupil diameter may reflect affective involvement beyond a simple stimulus response. In parallel, work in content analysis, advertising, and interface research has used activation in the DLPFC as a metric of consumers' cognitive engagement (Gier et al., 2020).

Accordingly, this study employed an eye-tracker and fNIRS in parallel to integratively evaluate users' visual and neurophysiological responses across age conditions and image type conditions. With the eye-tracker, we observe the affective responses elicited by each stimulus and the ensuing positive acceptance, and with fNIRS, we analyze condition-specific HbO activation patterns in the prefrontal cortex to assess the level of neural responses related to higher-order cognitive control, evaluation and decision-making. Beyond this, by combining and interpreting the two physiological metrics, this integrated approach aims to objectively clarify the linkage between character-type features and user responses and, ultimately, to propose design directions for AI-Generated Characters that are appropriate for the content context.

## 2 Methods

### 2.1 Participants

This study recruited healthy adult students (aged 21–26 years) from Hongik University through postings on campus online communities, and eligibility was screened using a pre-study questionnaire. The questionnaire explicitly stated that only right-handed individuals were eligible to participate, and it additionally assessed neurological or psychiatric history and current use of medications that could affect central nervous system or brain function; participants were enrolled only if they met these eligibility requirements. Because eye-tracking and fNIRS data were collected concurrently, pre-measurement calibration/quality checks were conducted for both devices to ensure data reliability. Participants were excluded from analysis if gaze tracking was not sufficiently stable or if the fNIRS calibration/quality check indicated insufficient channel-level signal or unstable measurements.

A total of 24 participants met the eligibility criteria and completed the experiment (21–26 years; 11 males, 45.8%; 13 females, 54.2%; all right-handed). Prior to participation, all individuals received a full explanation of the study purpose and procedures, as well as potential mental fatigue and discomfort during the session, and provided written informed consent. Importantly, participants were informed in advance that the stimulus set included both real facial photographs and AI-generated character images, and they participated with full awareness of these image types. The study was conducted in accordance with the Declaration of Helsinki and was approved by the Institutional Review Board of Hongik University (IRB no. 7002340-202409-HR-023).

### 2.2 Visual stimuli

This study used static facial images created by crossing the age conditions with the image type conditions. The six age conditions were infant, child, adolescent, adult, middle-aged, and elderly, and the three image type conditions were real, two-dimensional (2D), and three-dimensional (3D). One image was prepared for each combination, yielding 18 images in total.

Real stimuli were sourced from licensed stock image libraries, and portrait rights and secondary-use permissions were verified. To reduce unnecessary visual differences across conditions, only images that satisfied all of the following criteria were selected and used: (i) overall illumination and contrast were not overly biased, (ii) face and gaze direction were front-facing, and (iii) facial expression showed a subtle, non-exaggerated, emotionally neutral smile. After selection, the original background was removed and replaced with a uniform white background to standardize background complexity across real stimuli. In addition, all real stimuli had sufficient resolution, and a 4:5 face guide was applied to standardize face-to-frame proportion and align key facial landmarks, thereby minimizing differences in visual salience across real stimuli.

AI-generated character stimuli were produced using MidJourney. For each age group, the selected real photograph was uploaded as a reference image, and prompts were constructed to ensure that the same key factors applied in the real-stimulus selection stage (illumination/contrast, front-facing face and gaze direction, a natural non-exaggerated smiling expression, and a uniform white background) were maintained, thereby reducing the possibility of bias toward a specific image type due to perceptual mismatch between the real and AI stimuli. In addition, generation was guided using reference-image prompting and a consistent 4:5 aspect ratio to preserve structural correspondence with the reference photographs while producing type-specific outputs. After generation, all images were standardized using the same resolution, cropping, and background processing pipeline, and the mean luminance and contrast were computed within a predefined facial region-of-interest (ROI) to confirm that low-level visual properties were comparable across image types. The ROI-based verification confirmed that mean luminance and contrast values across the real, 2D, and 3D conditions fell within a comparable range, with no systematic directional bias toward any specific image type. Nevertheless, to prevent the inherent visual characteristics of each image type from being compromised, the 2D stimuli were guided to retain illustration-like features based on minimal line work and flat colors and shading, whereas the 3D stimuli were guided by adjusting material rendering and lighting-based shading so that three-dimensional depth was naturally expressed. Through this process, structural correspondence with the real photographs was maintained while the intended representational characteristics of each image type were clearly differentiated.

**Figure 1:**
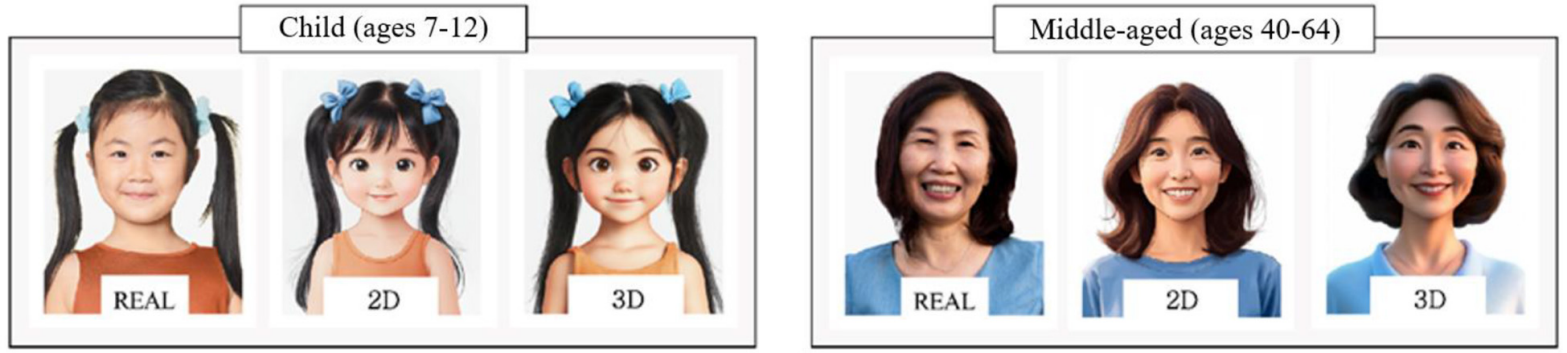
Visual stimuli used in the experiment: Real, 2D, and 3D images of infant, child, adolescent, adult, middle-aged, and elderly. This study primarily focused on the child and middle-aged conditions as the key comparison for analysis. Real, Realistic images; 2D, Two-dimensional cartoon images; 3D, Three-dimensional character images; Infant, 0–6 years; Child, 7–12 years; Adolescent, 13–19 years; Adult, 20–39 years; Middle-aged, 40–64 years; Elderly, 65 years and above. Photographic source images designed by Freepik. 2D and 3D images were generated using Midjourney Version 6.1.

To evaluate whether the AI-generated candidates were not excessively dissimilar from the reference real photographs (realism) and whether the intended visual characteristics of each image type (2D vs. 3D) were appropriately implemented, a panel of five experts with expertise in character visualization, digital character design, and visual image-based content production reviewed the generated candidates. The panel rated each candidate on a 5-point Likert scale for (i) naturalness and realism relative to the reference real photographs, (ii) appropriateness of the implementation of type-specific visual characteristics, and (iii) absence of unnecessary visual differences that could compromise fairness of comparisons across types. Based on these ratings, the final AI-generated stimuli were selected by choosing the candidate with the highest overall mean score within each age-by-type combination, thereby maintaining correspondence with the reference photographs while appropriately reflecting the intended visual characteristics of each image type (Figure 1).

### 2.3 Experimental design

**Figure 2:**
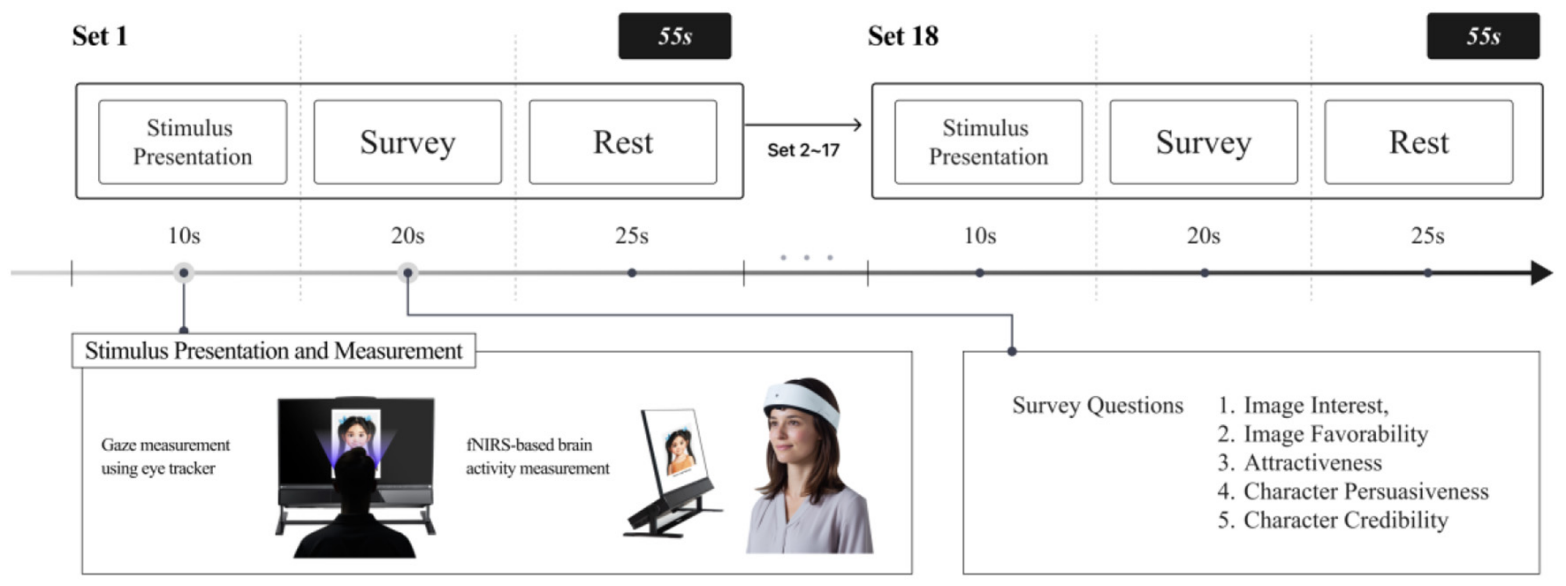
Experiment design. Adapted with permission from the OBELAB fNIRS device reference image by OBELAB Inc., licensed for use with attribution. Source: OBELAB homepage (www.obelab.com). Photographic source images designed by Freepik.

Each participant was presented with image stimuli divided by age conditions and image type conditions in randomized order. Each stimulus appeared on the screen for 10 s, during which participants freely observed it. Thereafter, participants completed a questionnaire regarding the stimulus. One set comprised 10 s of stimulus observation, 20 s of questionnaire response, and 25 s of rest, totaling 55 s per set. Prior to the session, participants were explicitly instructed to minimize head and body movements throughout the task to reduce motion-related artifacts in the eye-tracking and fNIRS recordings. All participants completed 18 sets following the same procedure, and the entire session lasted approximately 16 min and 30 s. An eye-tracker and fNIRS were used to measure gaze responses and cerebral hemodynamic changes, respectively (Figure 2).

### 2.4 Data acquisition

#### 2.4.1 Questionnaire

The survey comprised five 1–5 Likert-scale items, administered after each stimulus under the age conditions and image type conditions. To estimate image interest, we asked how strongly the stimulus captured and maintained visual attention. To estimate image liking, we asked how positively the stimulus was felt overall. To estimate attractiveness, we asked how aesthetically appealing the stimulus appeared with respect to form, color, and composition. To estimate character persuasiveness, we asked how convincing the character's message or intent was and the extent to which participants were willing to accept it. To estimate character trustworthiness, we asked how reliable, honest, and consistent the character appeared as an information source or social agent. Responses were recorded on-screen using a 5-point scale where 1 indicated not at all and 5 indicated very much. For analysis, item scores were summarized per participant within each age conditions and image type conditions.

#### 2.4.2 Eye-tracker data

The eye-tracker is a noninvasive device that records eye movements in real time to infer visual attention and cognitive processing, outputting basic metrics such as fixations, saccades, and pupil diameter. Here, a fixation refers to the duration and count of gaze remaining at one location, and a saccade refers to the rapid movement of gaze from one fixation point to another. These metrics are used to quantify gaze behavior toward visual stimuli (Duchowski, 2017). This study used a Tobii Pro Spectrum (Tobii Technology AB, Stockholm, Sweden). The device operates by illuminating the eyes with near-infrared light and estimating eye movements from the relative positions of the pupil center and corneal reflection captured by the camera. The maximum sampling rate was set to 1,200 Hz. To quantitatively analyze participants' visual attention to the stimuli, we used the following four eye-tracker metrics as dependent variables:

(1) Total duration of fixations—the sum of all fixation durations within each Area of Interest (AOI), indexing the overall maintenance of visual attention to a given stimulus or region. A longer total duration indicates more sustained visual interest or a higher cognitive processing load. (2) Average duration of fixations—the mean duration of individual fixations within each AOI, reflecting the depth of cognitive operations involved in interpreting or assigning meaning to visual information. Longer average durations imply that more cognitive resources are being allocated to information processing. (3) Number of fixations—the total count of fixations occurring within each AOI, capturing the frequency and strategy of visual exploration. A larger number of fixations can indicate finer-grained information search or greater complexity of the target of interest (Holmqvist et al., 2011). (4) Average pupil diameter—the mean pupil diameter measured within each AOI, used as a physiological metric of emotional arousal or cognitive load. Larger pupils are interpreted as indicating higher emotional arousal or greater attentional demand to the stimulus (Bradley et al., 2008).

#### 2.4.3 fNIRS data

**Figure 3:**
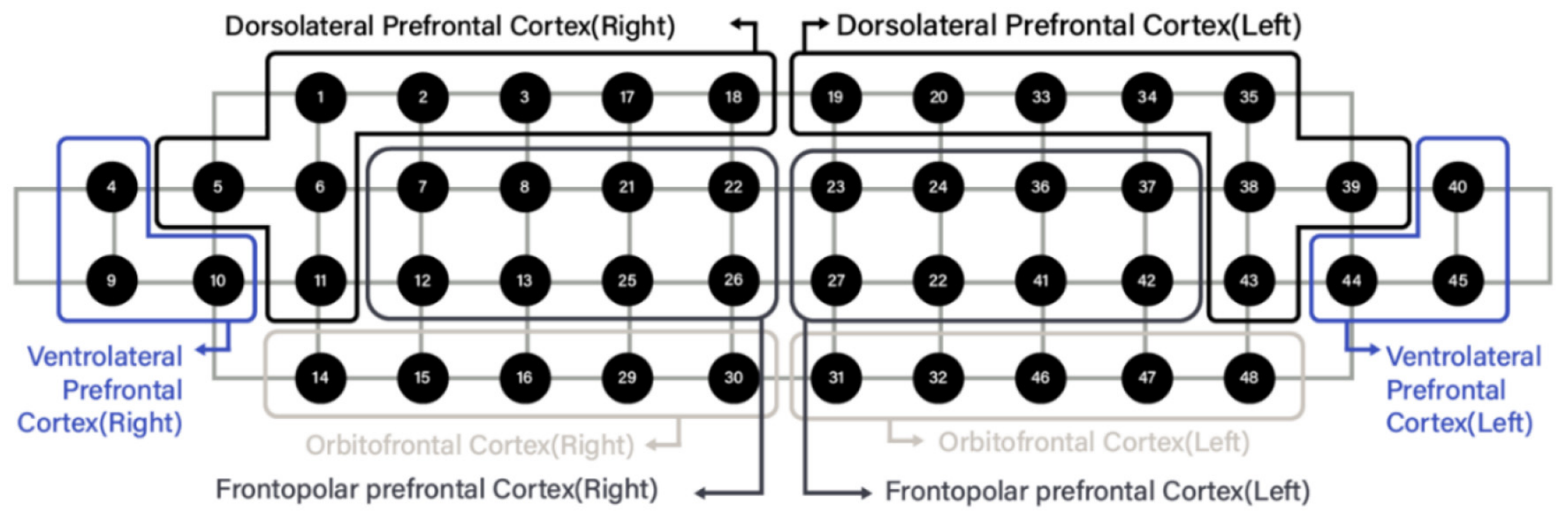
Configuration and measurement positions of the 48-channel NIRSIT fNIRS system. fNIRS channels were assigned to cortical subregions according to the default NIRSIT montage, providing 48 prefrontal channels grouped as follows: the right DLPFC encompassed channels 1, 2, 3, 5, 6, 11, 17, and 18; the left DLPFC, channels 19, 20, 33, 34, 35, 38, 39, and 43; the right Frontopolar Prefrontal Cortex (FPFC), channels 7, 8, 12, 13, 21, 22, 25, and 26; the left FPFC, channels 23, 24, 27, 28, 36, 37, 41, and 42; the right Orbitofrontal Cortex (OFC), channels 14, 15, 16, 29, and 30; the left OFC, channels 31, 32, 46, 47, and 48; the right Ventrolateral Prefrontal Cortex (VLPFC), channels 4, 9, and 10; and the left VLPFC, channels 40, 44, and 45.

fNIRS is a noninvasive technique that illuminates the scalp with near-infrared light and quantifies changes in oxygenated hemoglobin (HbO) concentration associated with cerebral hemodynamics (Scholkmann et al., 2014). In this study, prefrontal HbO responses during task performance were recorded using NIRSIT (OBELAB Inc., Seoul, Republic of Korea). The system uses two wavelengths (780 and 850 nm) and simultaneously acquires signals from 48 channels over the prefrontal cortex. The probe was positioned according to the default NIRSIT montage to provide consistent prefrontal coverage across participants, and channel-to-region assignments followed the manufacturer's default mapping (Figure 3).

Data were processed using NIRSIT Quest (v1.2.3.5) following the software's preprocessing pipeline. Prior to preprocessing, raw intensity data were inspected for invalid values. When invalid values occurred in runs of no more than five consecutive samples, nearest-neighbor interpolation was applied; channels were rejected when more than five consecutive invalid samples were present. Channel-level quality control was then performed at the raw intensity level. Specifically, channels were rejected if the median intensity was < 30, if the coefficient of variation exceeded 15% (Pfeifer et al., 2018), or if saturation was detected (i.e., identical consecutive values comprising >5% of the entire time series). After quality control, light intensity signals were converted to optical density (Yücel et al., 2021). Head motion can induce motion artifacts by causing optode displacement relative to the head, which can contaminate the measured fNIRS signal (Al-Omairi et al., 2023). Motion artifacts were corrected on the optical density time series using Temporal Derivative Distribution Repair (TDDR; Fishburn et al., 2019). Optical density data were then converted to hemoglobin concentration changes *via* the modified Beer–Lambert law (Cope et al., 1988; Delpy et al., 1988). Following ISO guidance within the Quest workflow, concentration changes were computed without applying a differential pathlength factor, and molar extinction coefficients were based on the dataset reported by Zhao et al. (2017). At the hemoglobin level, channels were additionally rejected when HbO demonstrated extreme negative correlation ( ≤ −0.9), consistent with mirroring-type artifact patterns (Takizawa et al., 2014).

The HbO time series was then digitally filtered using a DCT bandpass filter (0.005–0.1 Hz) to remove slow drift and high-frequency physiological or measurement noise (Yücel et al., 2021). When rejected channels required padding within the Quest workflow, their values were replaced using backup channels with matched distance and target location, or by the mean values of the corresponding Brodmann-area group, as implemented in NIRSIT Quest (v1.2.3.5).

### 2.5 Statistical analysis

All statistical analyses were performed in Stata/BE, version 19.5 (StataCorp LLC, College Station, TX, USA). The nominal alpha level was set at *p* < 0.05 (two-tailed). Descriptive statistics are reported as Mean ± SD.

#### 2.5.1 Experimental factors and primary inference framework

Because each participant viewed all 18 stimuli, all outcomes were analyzed using within-subject (repeated-measures) models with two fixed within-subject factors: Age (6 levels: infant, child, adolescent, adult, middle-aged, and elderly) and Image Type (3 levels: real, 2D, 3D). For each dependent variable, the primary omnibus test was a 6 × 3 repeated-measures ANOVA testing (i) the main effect of Age, (ii) the main effect of Image Type, and (iii) the Age × Image Type interaction. This framework was applied to the four eye-tracking outcomes (total duration of fixations, average duration of fixations, number of fixations, and average pupil diameter) and to fNIRS outcomes (mean HbO change) computed separately for each of the 48 channels.

#### 2.5.2 Assumption checks and corrections

Normality within each condition was examined using skewness and kurtosis. Sphericity was assessed with Mauchly's test. When sphericity was violated (*p* < 0.05), Greenhouse–Geisser–corrected degrees of freedom were used to preserve valid inference (Greenhouse and Geisser, 1959; Mauchly, 1940).

#### 2.5.3 Follow-up tests and multiple-comparison control

When the omnibus ANOVA indicated a significant main effect or interaction, follow-up analyses were conducted to localize the source of the effect. If a significant Age × Image Type interaction was observed, simple main-effects analyses were performed (Age effects within each Image Type level and/or Image Type effects within each Age level), followed by paired-samples comparisons where appropriate. Familywise error for *post hoc* pairwise comparisons was controlled using Bonferroni adjustment. For fNIRS, channel-wise mean HbO change was computed from block-averaged HbO responses during the 10-s stimulus period relative to the immediately preceding rest baseline. Short-separation regression (SSR; channels ≤ 15 mm) was applied to reduce superficial physiological noise (Vidal-Rosas et al., 2021). Statistical inference was conducted channel-wise; *p*-values from the 48 channel-wise omnibus tests were adjusted using the false discovery rate (FDR). *Post hoc* comparisons were interpreted only for channels meeting this criterion.

#### 2.5.4 Effect size reporting and calculation

In addition to *F* and *p* values, partial eta squared (ηp^2^) was reported as the effect-size index for all repeated-measures ANOVA effects. When not directly provided by the software output, ηp^2^ was computed using ηp^2^ = (F × df_n) / (F × df_n + df_d). Effect-size benchmarks: < 0.01 small; 0.01–0.06 small-medium; 0.06–0.14 medium; >0.14 large.

## 3 Results

### 3.1 Questionnaire data

**Figure 4:**
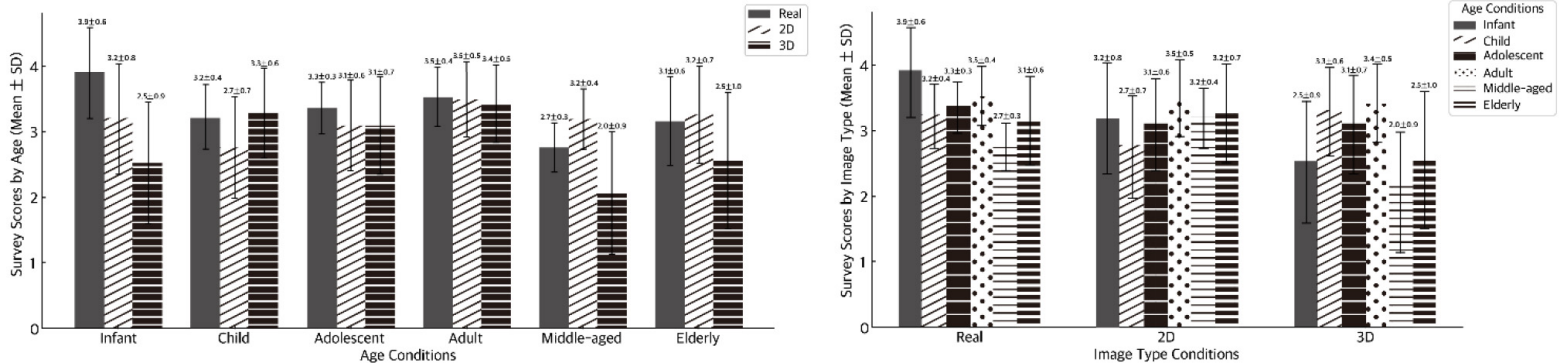
Comparison of socio-perceptual ratings across age and image type conditions. This Figure presents the mean and SD of five survey items, namely image interest, image liking, attractiveness, character persuasiveness, and character trustworthiness, rated on a 1–5 scale across the age conditions and image type conditions. Real, Realistic images; 2D, Two-dimensional cartoon images; 3D, Three-dimensional character images; Infant, 0–6 years; Child, 7–12 years; Adolescent, 13–19 years; Adult, 20–39 years; Middle-aged, 40–64 years; Elderly, 65 years and above.

Within the age conditions, the adult condition tended to be relatively higher than the other age conditions, and the infant condition under the real image type showed a notably higher mean. Within the image type conditions, scores were generally lower in 3D. These outcomes are descriptive; no inferential tests were applied to the questionnaire metrics (Figure 4).

### 3.2 Eye-tracker

We compared total duration of fixations, average duration of fixations, number of fixations, and average pupil diameter across the age conditions and the image type conditions. The analyses showed significant differences in number of fixations and average pupil diameter. For average pupil diameter, a significant difference across the age conditions was observed within the 3D image type condition; the middle-aged condition differed significantly from the other age conditions (*p* < 0.05).

For average pupil diameter, a significant difference across the image type conditions was observed within the middle-aged condition; the 3D condition was significantly higher than the real and 2D conditions (*p* < 0.05). In addition, within the 3D image type condition, a significant difference across the age conditions was observed; the middle-aged condition differed significantly from the other age conditions (*p* < 0.05; Table 1).

**Table 1:** Effects of age conditions on visual attention.

Variable	Type	Infant (Mean ±SD)	Child (Mean ±SD)	Adolescent (Mean ±SD)	Adult (Mean ±SD)	Middle -aged (Mean ±SD)	Elderly (Mean ±SD)	*F*	*P*	ηp^2^
Total duration of fixations (ms)	Real	6,724.53 ± 2,617.46	6,568.29 ± 2,264.14	6,908.77 ± 1,812.70	5,863.88 ± 2,560.38	6,220.53 ± 2,686.97	7,170.06 ± 1,388.77	0.74	0.597	0.044
	2D	6,865.12 ± 2,256.70	7,055.88 ± 1,234.61	6,807.41 ± 2,132.63	7,100.06 ± 1,264.38	6,581.24 ± 2,246.15	6,402.47 ± 2,627.10	0.3	0.911	0.018
	3D	5,911.59 ± 2,817.36	7,245.65 ± 1,348.92	6,632.53 ± 2,221.41	6,940.82 ± 1,645.99	7,335.47 ± 1,558.03	6,611.35 ± 2,361.45	1.09	0.372	0.064
Average duration of fixations (ms)	Real	260.19 ± 54.27	225.38 ± 33.01	244.82 ± 53.13	254.81 ± 76.71	268.88 ± 99.83	261.65 ± 42.59	0.96	0.444	0.074
	2D	283.71 ± 228.11	246.76 ± 49.01	242.50 ± 61.34	245.71 ± 46.73	240.18 ± 61.79	286.75 ± 186.49	0.47	0.800	0.035
	3D	224.06 ± 56.43	244.71 ± 40.11	246.38 ± 62.53	238.71 ± 52.86	241.18 ± 53.78	242.81 ± 49.31	0.38	0.863	0.037
Number of fixations (count)	Real	25.41 ± 8.52	28.94 ± 8.91	28.18 ± 6.00	23.24 ± 9.67	24.00 ± 10.27	27.94 ± 6.23	1.37	0.241	0.079
	2D	27.35 ± 9.84	29.06 ± 5.27	29.00 ± 9.76	29.41 ± 5.94	27.00 ± 8.22	24.76 ± 10.50	0.74	0.597	0.044
	3D	25.59 ± 11.07	29.71 ± 4.67	27.47 ± 8.71	29.29 ± 6.35	30.76 ± 6.28	27.24 ± 9.57	0.95	0.454	0.056
Average pupil diameter (mm)	Real	3.26 ± 0.44	3.13 ± 0.42	3.30 ± 0.44	3.15 ± 0.48	3.26 ± 0.41	3.07 ± 0.44	0.67	0.646	0.053
	2D	3.22 ± 0.45	3.24 ± 0.47	3.23 ± 0.47	3.19 ± 0.47	3.25 ± 0.42	2.96 ± 0.35	1	0.424	0.071
	3D	3.19 ± 0.39^a^	3.35 ± 0.48^b, c^	3.30 ± 0.49^d^	3.23 ± 0.46^e^	3.70 ± 0.66^a, b, d, e, f^	2.96 ± 0.45^c, f^	3.88^**^	0.003	0.230

Values are presented as Mean ± SD. Real, realistic images; 2D, two-dimensional cartoon images; 3D, three-dimensional character images; Infant, 0–6 years; Child, 7–12 years; Adolescent, 13–19 years; Adult, 20–39 years; Middle-aged, 40–64 years; Elderly, 65 years and above; a, Infant vs. Middle-aged; b, Child vs. Middle-aged; c, Child vs Elderly; d, Adolescent vs Middle-aged; e, Adult vs Middle-aged; f, Middle-aged vs. Elderly; ms, millisecond; mm, millimeter; Statistical significance is indicated as follows: ^**^p < 0.01. Effect size interpretation for η*p*^2^ is as follows: < 0.01 small; 0.01–0.06 small-medium; 0.06–0.14 medium; >0.14 large.

For number of fixations, a significant difference across the image type conditions was observed within the adult condition; the real image type condition showed a significantly lower value compared to the 2D and 3D image type conditions (*p* < 0.05). For average pupil diameter, a significant difference across the image type conditions was observed within the middle-aged condition; the 3D image type condition showed a significantly different value compared to the real and 2D image type conditions (*p* < 0.05; Table 2).

**Table 2:** Effects of image type conditions on visual attention.

Variable	Age	Real (Mean ±SD)	2D (Mean ±SD)	3D (Mean ±SD)	*F*	*P*	ηp^2^
Total duration of fixations (ms)	Infant	6,724.53 ± 2,617.46	6,865.12 ± 2,256.70	5,911.59 ± 2,817.36	0.68	0.512	0.041
	Child	6,568.29 ± 2,264.14	7,055.88 ± 1,234.61	7,245.65 ± 1,348.92	0.74	0.485	0.044
	Adolescent	6,908.77 ± 1,812.70	6,807.41 ± 2,132.63	6,632.53 ± 2,221.41	0.08	0.925	0.005
	Adult	5,863.88 ± 2,560.38	7,100.06 ± 1,264.38	6,940.82 ± 1,645.99	2.12	0.131	0.117
	Middle-aged	6,220.53 ± 2,686.97	6,581.24 ± 2,246.15	7,335.47 ± 1,558.03	1.12	0.333	0.065
	Elderly	7,170.06 ± 1,388.77	6,402.47 ± 2,627.10	6,611.35 ± 2,361.45	0.56	0.576	0.034
Average duration of fixations (ms)	Infant	260.19 ± 54.27	283.71 ± 228.11	224.06 ± 56.43	0.74	0.484	0.058
	Child	225.38 ± 33.01	246.76 ± 49.01	244.71 ± 40.11	1.33	0.275	0.093
	Adolescent	244.82 ± 53.13	242.50 ± 61.34	246.38 ± 62.53	0.02	0.983	0.002
	Adult	254.81 ± 76.71	245.71 ± 46.73	238.71 ± 52.86	0.3	0.742	0.023
	Middle-aged	268.88 ± 99.83	240.18 ± 61.79	241.18 ± 53.78	0.79	0.459	0.067
	Elderly	261.65 ± 42.59	286.75 ± 186.49	242.81 ± 49.31	0.61	0.548	0.048
Number of fixations (count)	Infant	25.41 ± 8.52	27.35 ± 9.84	25.59 ± 11.07	0.2	0.818	0.012
	Child	28.94 ± 8.91	29.06 ± 5.27	29.71 ± 4.67	0.07	0.935	0.004
	Adolescent	28.18 ± 6.00	29.00 ± 9.76	27.47 ± 8.71	0.14	0.866	0.009
	Adult	23.24 ± 9.67^a, b^	29.41 ± 5.94^a^	29.29 ± 6.35^b^	3.76^*^	0.030	0.190
	Middle-aged	24.00 ± 10.27	27.00 ± 8.22	30.76 ± 6.28	2.76	0.074	0.147
	Elderly	27.94 ± 6.23	24.76 ± 10.50	27.24 ± 9.57	0.59	0.558	0.036
Average pupil diameter (mm)	Infant	3.26 ± 0.44	3.22 ± 0.45	3.19 ± 0.39	0.09	0.911	0.007
	Child	3.13 ± 0.42	3.24 ± 0.47	3.35 ± 0.48	0.98	0.384	0.065
	Adolescent	3.30 ± 0.44	3.23 ± 0.47	3.30 ± 0.49	0.1	0.908	0.007
	Adult	3.15 ± 0.48	3.19 ± 0.47	3.23 ± 0.46	0.13	0.880	0.010
	Middle-aged	3.26 ± 0.41^b^	3.25 ± 0.42^c^	3.70 ± 0.66^b, c^	4.17 ^*^	0.022	0.275
	Elderly	3.07 ± 0.44	2.96 ± 0.35	2.96 ± 0.45	0.4	0.674	0.032

Values are presented as Mean ± SD. Infant, 0–6 years; Child, 7–12 years; Adolescent, 13–19 years; Adult, 20–39 years; Middle-aged, 40–64 years; Elderly, 65 years and above; real, realistic images; 2D, two-dimensional cartoon images; 3D, three-dimensional character images; a, real vs. 2D; b, real vs. 3D; c, 2D vs. 3D; ms, millisecond; mm, millimeter; Statistical significance is indicated as follows: ^*^p < 0.05. Effect size interpretation for η*p*^2^ is as follows: < 0.01 small; 0.01–0.06 small-medium; 0.06–0.14 medium; >0.14 large.

**Figure 5:**
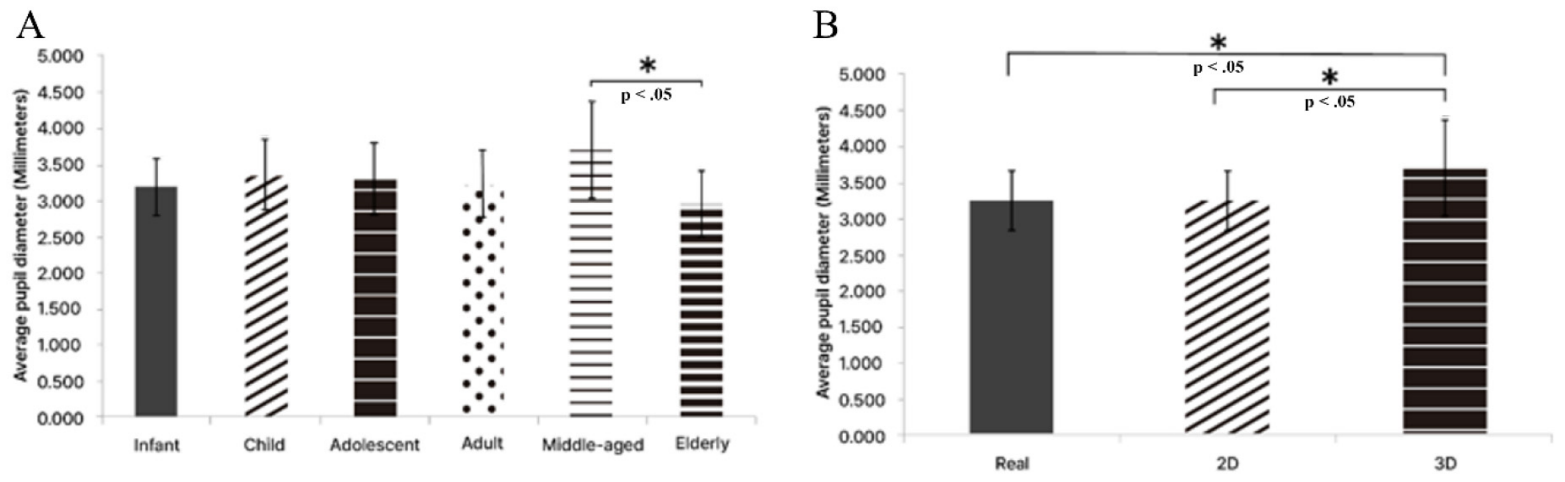
Main findings in eye-tracking: pupil diameter differences across age and image type. This Figure presents a core pattern in which the middle-aged condition combined with the 3D condition elicits the highest average pupil diameter relative to the other conditions. **(A)** Bar graph of average pupil diameter across age conditions. **(B)** Bar graph of average pupil diameter across image type conditions. Groups not sharing a letter are significantly different (*p* < 0.05). Asterisks indicate statistically significant or notable results. Infant, 0–6 years; Child, 7–12 years; Adolescent, 13–19 years; Adult, 20–39 years; Middle-aged, 40–64 years; Elderly, 65 years and above; Real, Realistic images; 2D, Two-dimensional cartoon images; 3D, Three-dimensional character images.

**Figure 6:**
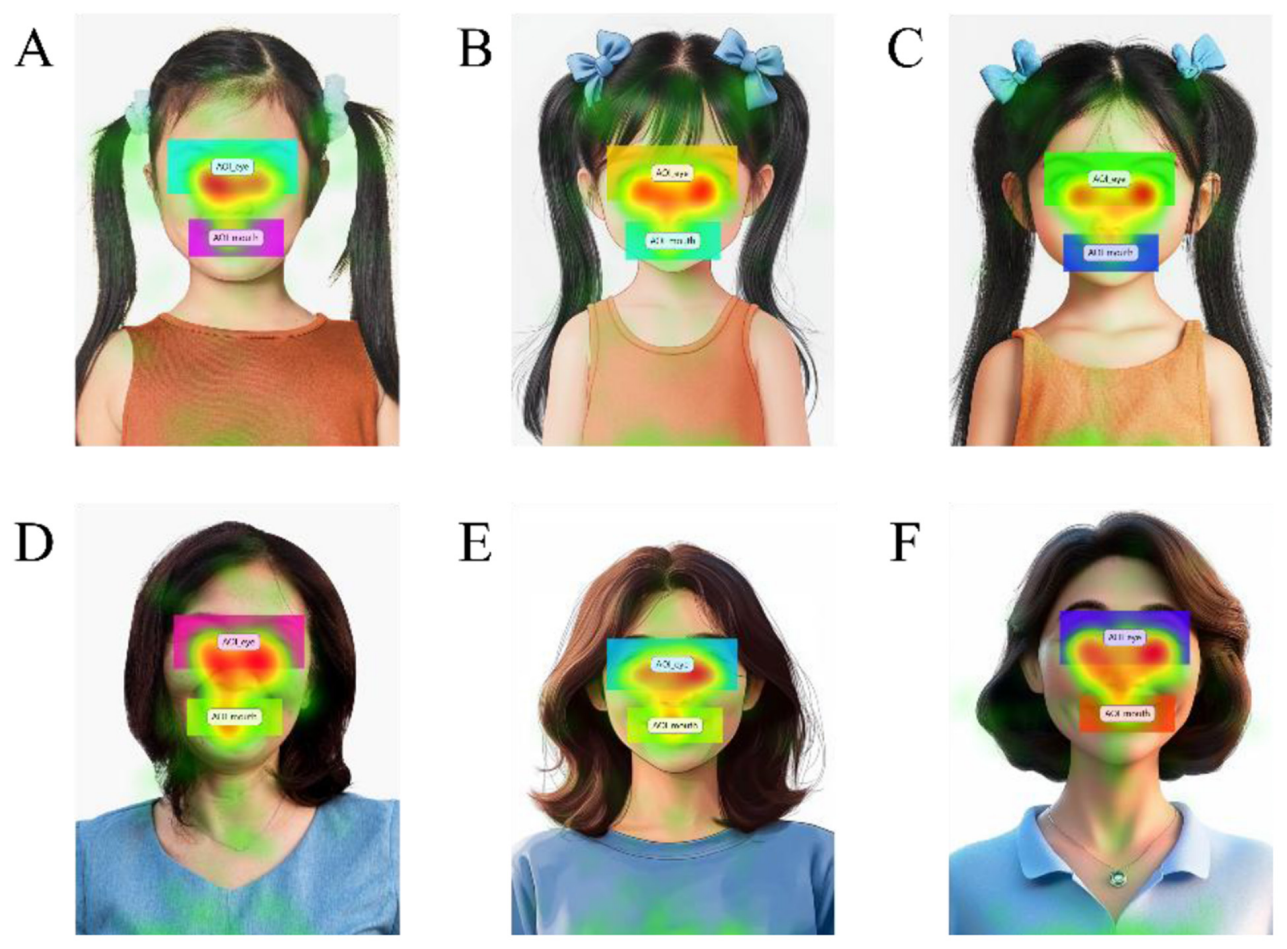
Eyes vs. mouth fixation heat maps for child and middle-aged faces across image types. This study primarily focused on the child and middle-aged conditions as the key comparison for analysis. The figure visualizes fixation-duration–weighted heat maps within two facial-feature AOIs (eyes vs. mouth). **(A)** Child–Real. **(B)** Child−2D. **(C)** Child−3D. **(D)** Middle-aged–Real. **(E)** Middle-aged−2D. **(F)** Middle-aged−3D. Heat maps represent the spatial accumulation of fixation duration, and the colored boxes delineate the predefined AOIs for the eyes and mouth used in feature-level analyses. Real, Realistic images; 2D, Two-dimensional cartoon images; 3D, Three-dimensional character images; Child, 7–12 years; Middle-aged, 40–64 years. Photographic source images designed by Freepik. 2D and 3D images were generated using Midjourney Version 6.1.

Within the adult condition, number of fixations showed a significant difference across the image type conditions (*p* = 0.0303, ηp^2^ = 0.190). The value in the real condition was significantly lower than in the 2D and 3D conditions. Within the 3D image type condition, average pupil diameter showed a significant difference across the age conditions (*p* = 0.0031, ηp^2^ = 0.230). The mean for the middle-aged condition was significantly higher than for the infant, child, adolescent, adult, and elderly conditions. Within the middle-aged condition, average pupil diameter showed a significant difference across the image type conditions (*p* = 0.0215, ηp^2^ = 0.275). The value in the 3D condition was significantly higher than in the real and 2D conditions. In line with the bar graphs, the middle-aged condition showed the largest average pupil diameter, and the elderly condition the smallest. The infant, child, adolescent, and adult conditions were intermediate. Across the image type conditions, the 3D condition was largest, whereas the 2D and real conditions were similar (Figures 5 and 6).

### 3.3 fNIRS

This study used one-way repeated-measures ANOVA to compare differences in HbO concentration across the age conditions and the image type conditions. Across the age conditions, significant differences were observed at Channel 5, Channel 19, and Channel 27 (*p* < 0.05). Overall, the child condition tended to differ from the other age conditions even at channels that did not reach significance, with the most pronounced separation at Channel 5. Across the image type conditions, widespread differences were evident in the left DLPFC, with a statistically significant effect at Channel 35 (*p* < 0.05). Several additional channels showed trend-level effects (*p* < 0.07; Table 3).

**Table 3:** Effects of age conditions on HbO concentration.

Region	Ch	Infant (Mean ±SD)	Child (Mean ±SD)	Adolescent (Mean ±SD)	Adult (Mean ±SD)	Middle-aged (Mean ±SD)	Elderly (Mean ±SD)	*F*	*P*	ηp^2^
DLPFC	Right DLPFC	3	0.001 ± 0.031	0.014 ± 0.036^e^	−0.008 ± 0.030^e, i, j^	0.013 ± 0.039^i^	0.008 ± 0.038^j^	0.002 ± 0.044	2.282	0.057	0.090
		5	−0.009 ± 0.028^a, d^	0.016 ± 0.035^a, e, f, g^	−0.005 ± 0.027^e^	−8.4771 × 10^−5^ ±0.030^f^	−0.002 ± 0.037^g^	0.006 ± 0.030^d^	3.936^**^	0.003	0.146
		18	−0.002 ± 0.033^a^	0.016 ± 0.036^a, g^	0.008 ± 0.031	0.009 ± 0.034	−0.001 ± 0.035^g^	0.004 ± 0.034	2.152	0.077	0.086
	Left DLPFC	19	−0.005 ± 0.026^a^	0.015 ± 0.036^a, e, f, g, h^	0.001 ± 0.029^e^	−0.004 ± 0.033^f^	−1.05771 × 10^−4^ ± 0.030^g^	−0.003 ± 0.035^h^	2.568^*^	0.038	0.100
		20	−0.003 ± 0.033^a^	0.012 ± 0.026^a, f, g^	0.009 ± 0.033	−0.002 ± 0.040^f^	−0.002 ± 0.036^g^	0.001 ± 0.036	1.641	0.163	0.067
		35	−4.61001 × 10^−4^ ± 0.026^a^	0.013 ± 0.034^af^	0.004 ± 0.035	−0.002 ± 0.038^f^	0.011 ± 0.035	0.008 ± 0.034	1.701	0.145	0.069
FPFC	Right FPFC	7	−0.005 ± 0.034^a^	0.017 ± 0.039^a, f, g^	0.006 ± 0.028	−0.001 ± 0.042^f^	1.55551 × 10^−4^ ± 0.031^g^	0.002 ± 0.031	2.346	0.052	0.093
		8	0.003 ± 0.020	0.010 ± 0.039	0.004 ± 0.023	0.009 ± 0.019	−1.52111 × 10^−4^ ± 0.024	−2.07111 × 10^−4^ ± 0.027	1.068	0.37	0.044
		21	−0.009 ± 0.028^a^	0.006 ± 0.035^a^	0.003 ± 0.031	−0.003 ± 0.032	−0.006 ± 0.031	−0.002 ± 0.036	1.292	0.272	0.053
	Left FPFC	27	−0.004 ± 0.033^a, b^	0.010 ± 0.032^a, g^	0.004 ± 0.028	0.013 ± 0.028^b, k, l^	−0.008 ± 0.034^g, k^	0.0007 ± 0.034^l^	3.143 ^*^	0.015	0.120
		36	−0.004 ± 0.039^b^	0.012 ± 0.044	0.008 ± 0.034	0.014 ± 0.036^b^	8.29771 × 10^−4^ ± 0.036	0.004 ± 0.036	1.492	0.203	0.061
		42	0.026 ± 0.044^b, c^	0.018 ± 0.042^g^	0.018 ± 0.037^j^	0.009 ± 0.049^b^	−1.72551 × 10^−4^ ± 0.038^c, g, j^	0.014 ± 0.045	2.212	0.068	0.088
VLPFC	Right VLPFC	4	−0.001 ± 0.022^a^	0.012 ± 0.027^a, e, g^	−0.001 ± 0.022^e^	0.002 ± 0.025	6.26111 × 10^−4^ ± 0.025^g^	0.008 ± 0.025	2.216	0.065	0.088
		9	−0.001 ± 0.022^a^	0.012 ± 0.027^a, e, g^	−0.001 ± 0.022^e^	0.002 ± 0.025	6.26111 × 10^−4^ ± 0.025^g^	0.008 ± 0.025	2.216	0.065	0.088
		10	−0.001 ± 0.022^a^	0.012 ± 0.027^a, e, g^	−0.001 ± 0.022^e^	−0.001 ± 0.022	6.26111 × 10^−4^ ± 0.025^g^	0.008 ± 0.025	2.216	0.065	0.088
	Left VLPFC	40	−3.12221 × 10^−6^ ± 0.020^a^	0.010 ± 0.021^ae^	3.44991 × 10^−4^ ± 0.015^e^	0.003 ± 0.022	0.002 ± 0.023	0.004 ± 0.024	1.403	0.235	0.057
		44	−3.12221 × 10^−6^ ± 0.020^a^	0.010 ± 0.021^a, e^	3.44991 × 10^−4^ ± 0.015^e^	0.003 ± 0.022	0.002 ± 0.023	0.004 ± 0.024	1.403	0.235	0.057
		45	−3.12221 × 10^−6^ ± 0.020^a^	0.010 ± 0.021^a, e^	3.44991 × 10^−4^ ± 0.015^e^	0.003 ± 0.022	0.002 ± 0.023	0.004 ± 0.024	1.403	0.235	0.057

Values are presented as Mean ± SD. HbO, oxygenated hemoglobin; DLPFC, dorsolateral prefrontal cortex; FPFC, frontopolar prefrontal cortex; VLPFC, ventrolateral prefrontal cortex; Ch, channel; Infant, 0–6 years; Child, 7–12 years; Adolescent, 13–19 years; Adult, 20–39 years; Middle-aged, 40–64 years; Elderly, 65 years and above; a, Infant vs. Child; b, Infant vs. Adult, c, Infant vs. Middle-aged; d, Infant vs. Elderly; e, Child vs. Adolescent; f, Child vs. Adult; g, Child vs. Middle-aged; h, Child vs. Elderly; i, Adolescent vs. Adult; j, Adolescent vs. Middle-aged; k, Adult vs. Middle-aged; l, Adult vs. Elderly; Statistical significance is indicated as follows: ^*^p < 0.05; ^**^p < 0.01. Effect size interpretation for ηp^2^ is as follows: < 0.01 small; 0.01–0.06 small-medium; 0.06–0.14 medium; >0.14 large.

**Figure 7:**
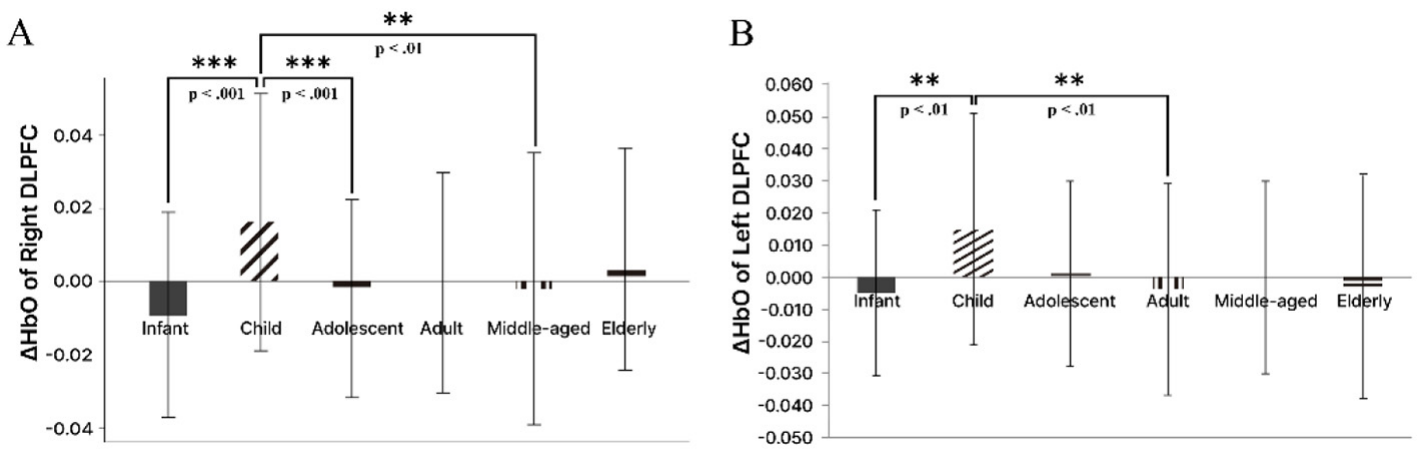
Differential fNIRS-measured HbO responses across age conditions: main comparisons. This Figure presents a core pattern in select DLPFC channels, where the child condition elicits the highest HbO responses overall relative to the other conditions. **(A)** Bar graph of HbO responses at Channel 5 in the right DLPFC. **(B)** Bar graph of HbO responses at Channel 19 in the left DLPFC. The y-axis shows mean HbO change from stimulus onset. Groups not sharing a letter are significantly different (*p* < 0.05). Asterisks denote *p* < 0.01. Infant, 0–6 years; Child, 7–12 years; Adolescent, 13–19 years; Adult, 20–39 years; Middle-aged, 40–64 years; Elderly, 65 years and above.

Within the age conditions, a significant difference emerged at Channel 5 in the right DLPFC (*p* = 0.003, ηp^2^ = 0.146). The child condition showed higher HbO than the infant, adolescent, adult, and middle-aged conditions. In particular, the contrasts with the infant and adolescent conditions were significant (*p* < 0.001) and remained significant after Bonferroni correction. The other pairwise differences did not survive correction. A significant difference also appeared at Channel 19 in the left DLPFC (*p* = 0.038, ηp^2^ = 0.100), where the child group showed higher HbO than the other age groups. However, this effect was not significant after correction. At Channel 27 in the left FPFC, a significant difference was observed (*p* = 0.015, ηp^2^ = 0.120). The child condition exceeded the infant and middle-aged conditions, and the adult condition exceeded the infant, middle-aged, and elderly conditions; none of these remained significant after correction. In the right Ventral Prefrontal Cortex (VLPFC), a similar trend was present without reaching significance (*p* = 0.065). Across the image type conditions, a significant difference was found at Channel 35 in the left DLPFC (*p* = 0.005, ηp^2^ = 0.190). The 2D and 3D conditions showed higher HbO than the real condition, and after correction only the real vs. 3D contrast remained significant. Channel 34 (*p* = 0.065) and Channel 39 (*p* = 0.062) showed trend-level differences (Figure 7).

## 4 Discussion

This study combined an eye-tracker and fNIRS to simultaneously quantify visual attention and engagement metrics together with prefrontal activation across the age conditions and the image type conditions. Analyses indicated that pairing the middle-aged condition with the 3D image type condition elicited a larger average pupil diameter. In fNIRS, increases in HbO at selected channels within the DLPFC were observed for the child condition and for the AI-Generated Characters condition, which may be compatible with greater prefrontal engagement under those stimulus properties; however, prefrontal HbO changes are not process-specific, and alternative interpretations cannot be ruled out. Taken together, jointly analyzing pupil responses and HbO changes provides an integrated account of how attention and overall prefrontal engagement vary with the age conditions and the image type conditions. Meanwhile, the questionnaire metrics were treated as secondary and summarized descriptively only, with mean ± SD reported by the age and image type conditions. No inferential tests were performed, and no theoretical claims were drawn from these metrics.

Within the middle-aged condition, the 3D image type condition elicited a relatively larger average pupil diameter. This pattern is unlikely to reflect only low-level visual factors; rather, it suggests that the stimulus elicited a degree of liking or a positive attitude. Moreover, pupil dilation is closely linked to emotional arousal, and stronger preference for a given target has been reported to accompany greater arousal and, correspondingly, larger pupil dilation (Bradley et al., 2008).

Beyond a simple correlation whereby pupil size increases when viewing preferred targets, recent work has begun to interpret pupil responses at the level of the content context. For example, when visual attention and pupil metrics were compared between video stimuli that did vs. did not evoke emotional rapport, peak pupil dilation was larger in the empathic condition, indicating greater narrative engagement and emotional immersion by viewers. Accordingly, the increase in average pupil diameter observed in this study suggests that, in content creation and analysis, one should consider not only liking but also context-level factors such as rapport and engagement (Zhang et al., 2022).

**Figure 8:**
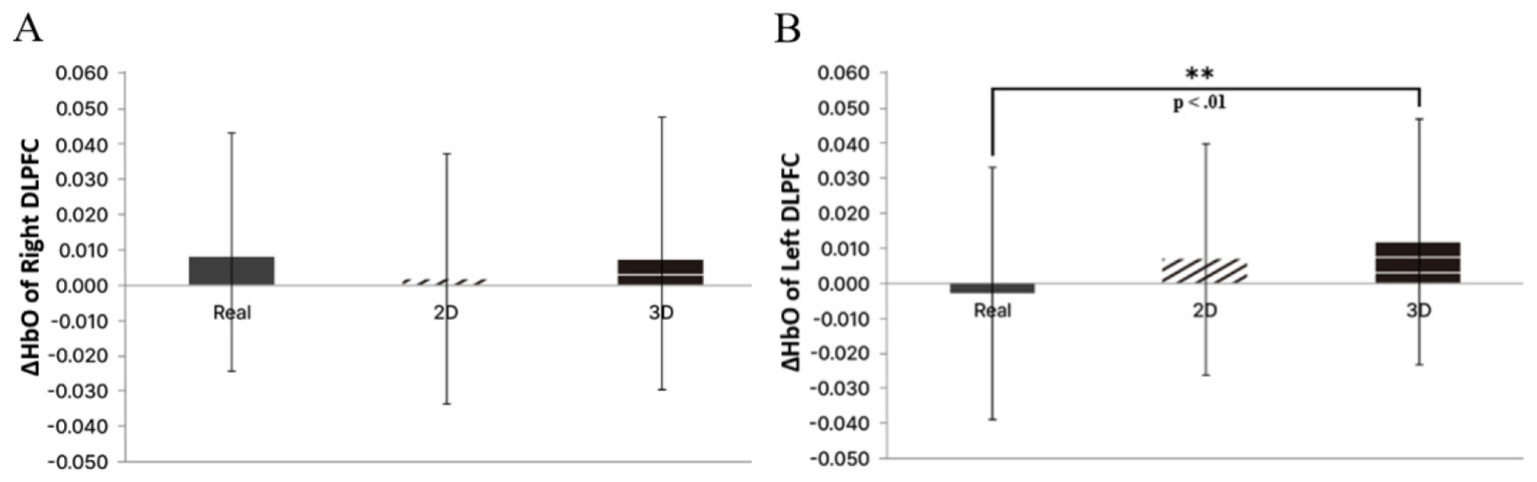
Key fNIRS-measured HbO differences across image type conditions. This Figure presents a core pattern in select left DLPFC channels, where the AI-Generated Characters condition elicits the highest HbO responses overall relative to the other conditions. **(A)** Bar graph of HbO responses at Channel 18 in the right DLPFC. **(B)** Bar graph of HbO responses at Channel 35 in the left DLPFC. The y-axis shows mean HbO change from stimulus onset. Groups not sharing a letter are significantly different (*p* < 0.05). Asterisks denote *p* < 0.01. Real, Realistic images; 2D, Two-dimensional cartoon images; 3D, Three-dimensional character images.

As summarized in Table 4, specific channels within the left DLPFC showed higher HbO in response to AI-Generated Characters, consistent with earlier results for the child condition, indicating that both conditions contribute to differences in prefrontal responses across conditions rather than implying a single underlying cognitive process. The association between the DLPFC and working memory has been well established by prior work. In particular, the DLPFC is more prominently engaged in higher-order working memory functions, including planning, organization, and strategic processing, than in simple maintenance (Kroger et al., 2002; Owen, 1997). Nevertheless, because DLPFC activity is engaged by multiple overlapping operations, these HbO changes are best interpreted cautiously as reflecting broader increases in cognitive demands, rather than improvement in a specific function such as working memory. In line with this interpretation, the HbO increases observed for the child condition and the AI-Generated Characters condition can be viewed as broadly consistent with relatively greater engagement of prefrontal resources during viewing (Figure 8).

**Table 4:** Effects of image type conditions on HbO concentration.

Region	Ch	Real (Mean ±SD)	2D (Mean ±SD)	3D (Mean ±SD)	*F*	*P*	ηp^2^
DLPFC	Right DLPFC	3	0.007 ± 0.039	0.006 ± 0.035	0.002 ± 0.045	0.477	0.615	0.020
		5	4.30551 × 10^−4^ ± 0.032	7.34001 × 10^−4^ ± 0.032	0.004 ± 0.034	0.383	0.666	0.016
		18	0.008 ± 0.033	0.001 ± 0.035	0.008 ± 0.038	1.401	0.249	0.057
	Left DLPFC	34	0.002 ± 0.040^b^	0.005 ± 0.035	0.014 ± 0.038^b^	2.773	0.065	0.108
		35	−0.003 ± 0.036^a, b^	0.007 ± 0.033^a^	0.012 ± 0.035^b^	5.388^**^	0.006	0.190
		39	−0.002 ± 0.038^b^	4.62771 × 10^−6^ ± 0.035	0.009 ± 0.038^b^	2.849	0.062	0.110
FPFC	Right FPFC	7	0.003 ± 0.034	0.008 ± 0.034	0.004 ± 0.036	1.04	0.353	0.043
		8	−0.001 ± 0.028	0.002 ± 0.030	0.006 ± 0.030	1.478	0.232	0.060
		21	0.003 ± 0.033	0.002 ± 0.037	−9.90551 × 10^−4^ ± 0.037	0.303	0.735	0.013
	Left FPFC	36	−7.68221 × 10^−5^ ± 0.035	0.007 ± 0.033	0.007 ± 0.041	1.278	0.28	0.053
		27	0.007 ± 0.034^a^	−0.003 ± 0.036^a^	0.004 ± 0.032	2.225	0.111	0.088
		42	0.012 ± 0.046	0.015 ± 0.045	0.014 ± 0.041	0.17	0.838	0.007
VLPFC	Right VLPFC	4	6.62881 × 10^−4^ ± 0.024	0.002 ± 0.023	0.001 ± 0.030	0.102	0.903	0.004
		9	6.62881 × 10^−4^ ± 0.024	0.002 ± 0.023	0.001 ± 0.030	0.102	0.903	0.004
		10	6.62881 × 10^−4^ ± 0.024	0.002 ± 0.023	0.001 ± 0.030	0.102	0.903	0.004
	Left VLPFC	40	0.002 ± 0.023	1.19001 × 10^−4^ ± 0.022	0.006 ± 0.022	2.01	0.137	0.080
		44	0.001 ± 0.022	9.03111 × 10^−5^ ± 0.022^c^	0.006 ± 0.022^c^	2.249	0.109	0.089
		45	9.88111 × 10^−4^ ± 0.022	−1.85441 × 10^−4^ ± 0.021	0.005 ± 0.022	1.618	0.201	0.066

Values are presented as Mean ± SD. HbO, oxygenated hemoglobin; DLPFC, dorsolateral prefrontal cortex; FPFC, frontopolar prefrontal cortex; VLPFC, ventrolateral prefrontal cortex; Ch, channel; Real, Realistic images; 2D, two-dimensional cartoon images; 3D, three-dimensional character images; a, real vs. 2D; b, real vs. 3D; c, 2D vs. 3D; Statistical significance is indicated as follows: ^**^p < 0.01. Effect size interpretation for η*p*^2^ is as follows: < 0.01 small; 0.01–0.06 small-medium; 0.06–0.14 medium; >0.14 large.

**Figure 9:**
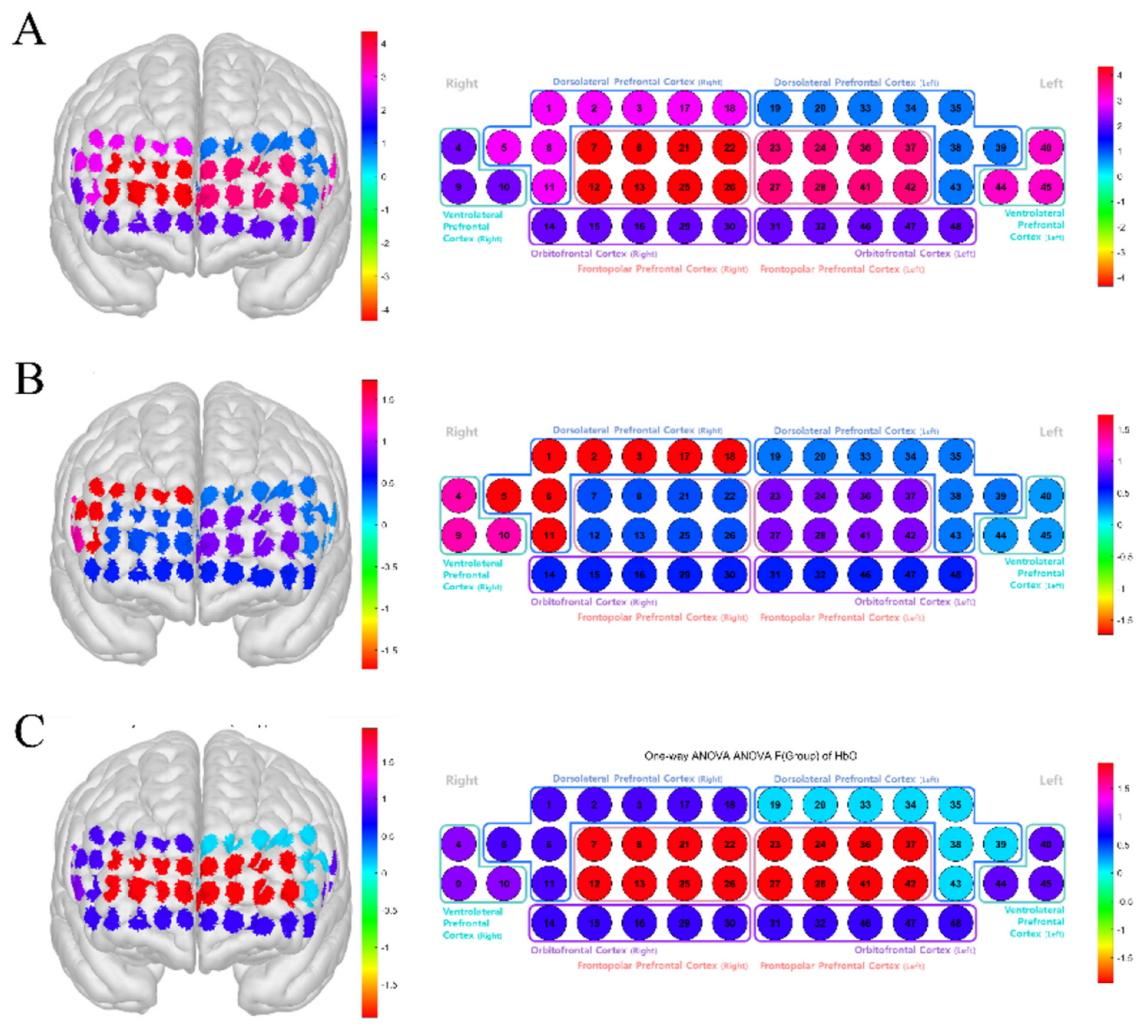
Channel-wise activation maps of HbO by stimulus condition (child, 2D, 3D). The figure visualizes channel-wise oxygenated hemoglobin (HbO) responses over the 48-channel prefrontal montage. **(A)** Child condition. **(B)** 2D condition. **(C)** 3D condition. For each panel, the left figure shows the three-dimensional cortical projection of the fNIRS channels, and the right figure shows the corresponding 2D schematic layout grouped by anatomical subregions and hemisphere. Channel colors represent the statistical values indicating the direction and magnitude of HbO responses for the displayed condition; color bars denote the value range. DLPFC, dorsolateral prefrontal cortex; VLPFC, ventrolateral prefrontal cortex; OFC, orbitofrontal cortex; FPFC, frontopolar prefrontal cortex; HbO, oxygenated hemoglobin; 2D, two-dimensional; 3D, three-dimensional.

Further, the importance of the DLPFC in self-control and impulse inhibition is well documented. Hare et al. (2009) showed that, in a food-choice task, value signals computed in the Ventromedial prefrontal cortex (vmPFC) enable health-conscious choices when modulated by a sector of the left DLPFC, rather than relying solely on immediate taste. Taken together with results from this study, stimuli depicting children and AI-Generated Characters could be discussed as potentially engaging prefrontal circuitry that has been implicated in regulatory control in prior work. However, based on the measures included in the present study, it remains difficult to directly substantiate whether these prefrontal responses reflect the selective recruitment of a specific regulatory process or translate into measurable changes in regulatory function (Figure 9).

However, the fact that stimuli featuring children and AI-Generated Characters showed higher HbO in certain subregions of the DLPFC does not, by itself, warrant the conclusion that working memory or emotion regulation improved. This caution aligns with the well-known issue of reverse inference (Poldrack, 2011). Activation observed in a portion of a given region may suggest possible involvement in a function, but it does not immediately imply improved behavioral performance. Recent studies indicate that increases in DLPFC activity do not necessarily translate into appetite suppression or enhanced self-control. Wilson et al. (2023) noted that, although DLPFC recruitment can aid dietary self-regulation, the likelihood of regulatory failure increases under sustained temptation or changing environments.

The combination of two physiological metrics observed in users—(1) average pupil diameter is markedly larger while DLPFC activation is lower, and (2) DLPFC activation is higher while average pupil diameter is smaller—suggests distinct cognitive states or processing modes. These patterns can be described as consistent with different processing modes, but they should not be treated as diagnostic because both pupil diameter and prefrontal HbO are influenced by multiple factors and do not map one-to-one onto a single psychological process.

First, when the pupils are dilated but DLPFC activation is low, this pattern can be interpreted as heightened emotional arousal with insufficient top-down cognitive control. In other words, attention is strongly pulled in a bottom-up manner by external stimuli while deliberate control remains limited (Mather and Sutherland, 2011). Potent affective stimuli reflexively dilate the pupils *via* sympathetic activation, whereas during passive viewing without an explicit regulation task, pronounced prefrontal engagement, particularly in the DLPFC, is often absent or reduced (Bradley et al., 2008; Buhle et al., 2014). Notably, under acute stress, increased norepinephrine release can produce rapid pupil dilation and engage affective regions such as the amygdala, while elevated arousal can weaken DLPFC function, leading to decrements in working memory and inhibitory control (Arnsten, 2009).

Second, when DLPFC activation is higher while average pupil diameter is smaller, this pattern can be interpreted as strong top-down cognitive control with low, stable autonomic arousal (Miller and Cohen, 2001). This profile often appears when the task is well learned or the stimulus is predictable or familiar, reducing processing load such that prefrontal goal maintenance and control persist without additional arousal, thereby limiting pupil dilation (Granholm and Steinhauer, 2004). In addition, during cognitive reappraisal, prefrontal activity, including the DLPFC, increases while subjective emotional arousal decreases, indicating that strengthened top-down control can suppress physiological arousal (Ochsner and Gross, 2005).

Taken together, jointly analyzing the two metrics yielded a more nuanced account of the interplay between bottom-up attentional capture and top-down cognitive control, consistent with the framework of Aston-Jones and Cohen (2005). This integrated perspective can reveal attentional-control mechanisms that might be missed when relying on a single metric. The complementary pattern observed between average pupil diameter and HbO across conditions indirectly suggests how users' cognitive engagement and physiological arousal vary with content attributes. Accordingly, combining an eye-tracker with fNIRS affords a deeper assessment of visual attention and cognitive state and offers practical guidance for optimizing content design. Building on this integrated interpretation, the observed patterns also allow tentative, design-relevant inferences regarding how different stimuli may shape users' viewing states.

In practical terms, the present pattern suggests that stimulus conditions linked to larger pupil dilation (e.g., the 3D middle-aged condition) may be more compatible with rapid attention capture and initial interest, whereas conditions linked to relatively higher DLPFC HbO (e.g., child and AI-generated character stimuli in select channels) may be compatible with greater evaluative demands that could warrant simpler, more digestible messaging. These applied interpretations remain tentative given the non-specificity of prefrontal HbO.

Despite these implications, several limitations should be considered when interpreting the findings of this study. First, the sample size (*n* = 24) and the demographic homogeneity of the participants (all adults in their 20s) limit the generalizability of the findings and may constrain statistical reliability. Second, because the sample consisted entirely of adults in their twenties, the study was not well positioned to detect differences across consumer age groups. Third, because the stimulus set comprised only female AI-generated characters, the ecological validity of the stimuli may be limited, and potential influences related to responses toward male characters or opposite-sex targets may not have been fully captured. Therefore, future research should examine whether the present results replicate across a wider range of conditions and contexts and test them in more expansive settings.

Accordingly, future research should recruit larger and more demographically diverse samples, include a broader range of stimulus identities (e.g., both female and male characters), and validate stimuli more extensively across contexts to strengthen generalizability and ecological validity. In addition, although the present study effectively combined eye-tracking and fNIRS, future work may benefit from integrating additional psychophysiological measures to more comprehensively characterize affective and cognitive processes, such as facial-expression analysis, electrodermal activity, and EEG. Finally, these design-oriented implications could be evaluated more directly by testing whether the observed physiological patterns translate into measurable user outcomes in applied settings, such as engagement, comprehension, and decision-related behavior.

## 5 Conclusion

This study suggests that, in future AI-Generated Characters and digital content production, tailored design strategies should reflect user characteristics by age and image type. By integrating an eye-tracker and fNIRS, we assessed visual attention and cognitive state from multiple angles. Considering the complementary relationship between the two physiological metrics, we found that users' emotional engagement and cognitive control vary with content characteristics. This integrated approach reveals attention-control mechanisms that may be missed when relying on a single metric and can be applied to the design of AI-Generated Characters and the evaluation of user experience. In particular, these findings can inform type-specific content development and strategies to optimize user engagement across education, entertainment, and marketing.

## Data Availability

The raw data supporting the conclusions of this article will be made available by the authors, without undue reservation.
